# Thermography as an aid for the complementary diagnosis of nodules in the thyroid gland

**DOI:** 10.1186/s12938-022-01009-3

**Published:** 2022-06-27

**Authors:** Viviane Magas Bittencourt de Camargo, Leandra Ulbricht, Jose Carlos Pereira Coninck, Wagner Luis Ripka, Humberto Remigio Gamba

**Affiliations:** 1 Federal Technological University of Paraná/Graduate Program in Electrical Engineering and Industrial Informatics, Endereço: Av. Sete de Setembro 3165. Campus Curitiba Sede Centro, Curitiba, PR CEP-80230-901 Brazil; 2 Federal Technological University of Paraná/Program in Biomedical Engineering, Endereço: Av. Silva Jardim, 807 - Bloco V3. Campus Curitiba Sede Centro, Curitiba, PR CEP-80230-901 Brasil; 3 Department of Statistics, Federal Technological University of Paraná/ Academic, Endereço: Av. Sete de Setembro 3165. Campus Curitiba Sede Centro, Curitiba, PR CEP-80230-901 Brasil

**Keywords:** Dynamic thermography, Thyroid nodule, Complementary diagnosis

## Abstract

**Background:**

Considering the estimate that thyroid cancer will become the fourth most prevalent type of tumor, improving its diagnosis is a necessity. The gold standard for evaluating thyroid nodules is ultrasound followed by biopsy. These tests, however, have limitations, especially in nodules smaller than 0.5 cm. Dynamic infrared thermography is an imaging method that does not require ionizing radiation or contrast injection. The aim of the study was to analyze the thermal behavior of thyroid nodules through infrared thermography using the cold stress protocol.

**Results:**

The Wilcoxon test showed thermal differences between groups (control and healthy, *p* < 0.001). The difference in the thermal behavior of the nodular tissues was evidenced by the longitudinal analysis. When comparing the nodules, it was possible to verify that the beginnings of tissue heating is significant (*p *= 0.001). In addition, the variability analysis showed a “well” effect, which occurred in period t-1 (pre-cooling time) to period *t* = 3 (time three minutes). Benign nodules had a variation ratio of 1.81 compared to malignant nodules.

**Conclusion:**

Benign nodules present a different thermal behavior than malignant nodules, and both present different behavior than normal tissue. For the analysis of nodules, the protocol used with cold stress, dynamic thermography and the inclusion of time t-1 were essential for the differentiation of nodules in the thyroid gland. Therefore, we recommend the continuance of these parameters for future studies.

**Methods:**

Thirty-three individuals with nodules in the thyroid region and nine healthy individuals participated in this descriptive exploratory study. In total, 42 nodules were evaluated, 11 malignant and 31 benign. The region of interest was exposed to cold stress for 30 s. First, the image was captured before the cold stress and subsequently, the images were assessed every 30 s, over a 10-min time period after cold stress. The perfusion and the thermal behavior of the tissues were evaluated by longitudinal analysis based on the number of pixels in each time period. The statistical tests of Wilcoxon, F-Snedecor and longitudinal models would assist in data analysis.

**Supplementary Information:**

The online version contains supplementary material available at 10.1186/s12938-022-01009-3.

## Background

Thyroid cancer is considered one of the most common endocrine malignant tumors, responsible for 3.4% of all cancers diagnosed annually. It occupies the eighth position in the ranking of cancers that affect women [1, 2]. Worldwide, this neoplasm affects, on average, 3% to 5% of all female cancers and 0.6% to 1.5% of male cancers [3, 4].

For early diagnosis, clinical screening is used (palpation of the nodule), image examination (usually ultrasound) and FNA (fine needle aspiration) [5]. These means, however, there are some limitations. Small nodules below 1 cm are barely palpable and nodules below 0.5 cm make it difficult to insert a needle into the biopsy and tend to generate a high rate of false positives [6, 7]. On the other hand, the scintigraphy exam, in addition to the cost which limits its use, emits radiation, implying a risk to the patient, especially in the case of pregnant women [8].

Despite being one of the most used methods for evaluating thyroid gland tumors, controversial results from some studies cast doubt on the real effectiveness of the ultrasound exam, showing a low sensitivity in the evaluation of thyroid carcinomas [9, 10].

Other less used tests also help in the diagnosis of thyroid nodules. Magnetic resonance imaging (MRI) with contrast and computed tomography (CT) are imaging exams that allow for a rigorous evaluation of the gland, and thus, enable a more accurate tracking of lesions [11, 12]. However, due to the high cost of the equipment and also the high price for the realization of the images, the feasibility for its use in poorer countries is reduced, as is the case of Brazil.

The limitations of the methods commonly used to identify and analyze thyroid gland nodules lead (Additional file 1: Table S1) the present research to suggest a new alternative to complementary imaging exams in assisting physicians in clinical diagnosis: the adoption of dynamic infrared thermography.

When carrying out the physiological analysis of neoplastic diseases, we see that nodules are overactive structures and they depend on blood vessels to obtain nutrients and oxygen, thus becoming highly vascularized structures [13, 14]. A neoplastic tissue is generally associated with increased local blood perfusion and increased metabolic rate, producing a change in skin temperature. This vascular growth is responsible for a relevant change in temperature in the nodule region which can be captured by thermal cameras [15, 16].

Research investigating the feasibility of thermography for the complementary diagnosis of thyroid cancer is still recent. In addition, some studies that have indicated an increase in temperature in diverse types of thyroid tumors have been identified [17–19]. When compared with conventional exams, such as ultrasonography, FNA and Doppler, the thermographic exam appears to be effective, with 100% sensitivity, surpassing the Doppler exam in the selection of thyroid nodules [18]. A more complex study with image processing conveyed a good percentage of accuracy for cancer diagnosis, calculated at around 78% to 89% [19].

Among the capturing techniques for thermography, dynamic stands out in the evaluation of thermal changes. In this technique, the region of interest (ROI) is exposed to thermal stress, and temperature changes are evaluated over time [13, 20]. In dynamic thermography, the exam is more reliable and less dependent on external agents, which is a relevant factor for the production of important physiological information regarding the region of interest [21].

The studies conducted have found good results in pointing out thermal changes in the region with nodules when compared to healthy tissue [18, 19]. However, the thermal difference between malignant and benign nodules and whether it is possible to differentiate the two types of nodules still represent a gap in the literature. Knowing the characteristics of the thermal behavior of regions with and without nodules, as well as the differences in this behavior, can enable the standardization of a thermal acquisition and analysis protocol for the characterization of nodules. Also, presenting one more resource for the evaluation of thyroid gland nodules contributes as a way to aid in complementary diagnosis, via a technology free of ionizing radiation, painless, non-invasive and easy to manage.

The objective of the study is to analyze the thermal behavior of the healthy and nodular thyroid region through dynamic infrared thermography after the application of cold stress.

## Results

### Temperature variation between tissues

**Fig. 1:**
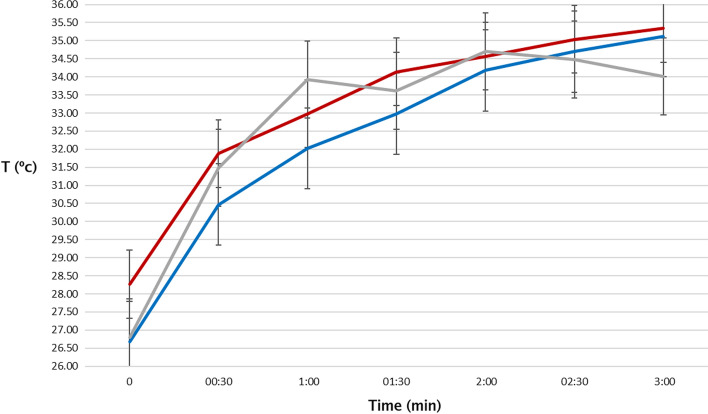
Median minimum temperatures of the malignant, benign nodules and the control group. The malignant nodule is represented by the red color, the benign ones by the blue color and the control group (healthy tissue) by the gray color. The figure shows the thermal difference (ºC) over time (minutes). Y-axis in degrees Celsius. Axis x time 3 min in a 30-s interval

In order to identify thermal asymmetry between the study group (malignant and benign nodules) and the control group, medians of minimum, average and maximum temperatures were evaluated. We observed (Fig. 1) that all median temperatures of malignant nodules were higher than median temperatures of benign nodules and higher than the control group (*p* < 0.001). On the other hand, the control group showed higher peaks than the study group at certain time intervals, with their temperatures decreasing considerably after a period of 2 min.

### Longitudinal data analysis

Aiming to analyze the thermal behavior of tissues over time, healthy tissues and tissues with nodules were studied right after the application of cold stress, in the period that goes from t_0 =_0 to 10 min(*t *= 10). For this, the random-effects model for longitudinal data (REM) was used and the differences between the groups were evaluated over time.

**Fig. 2:**
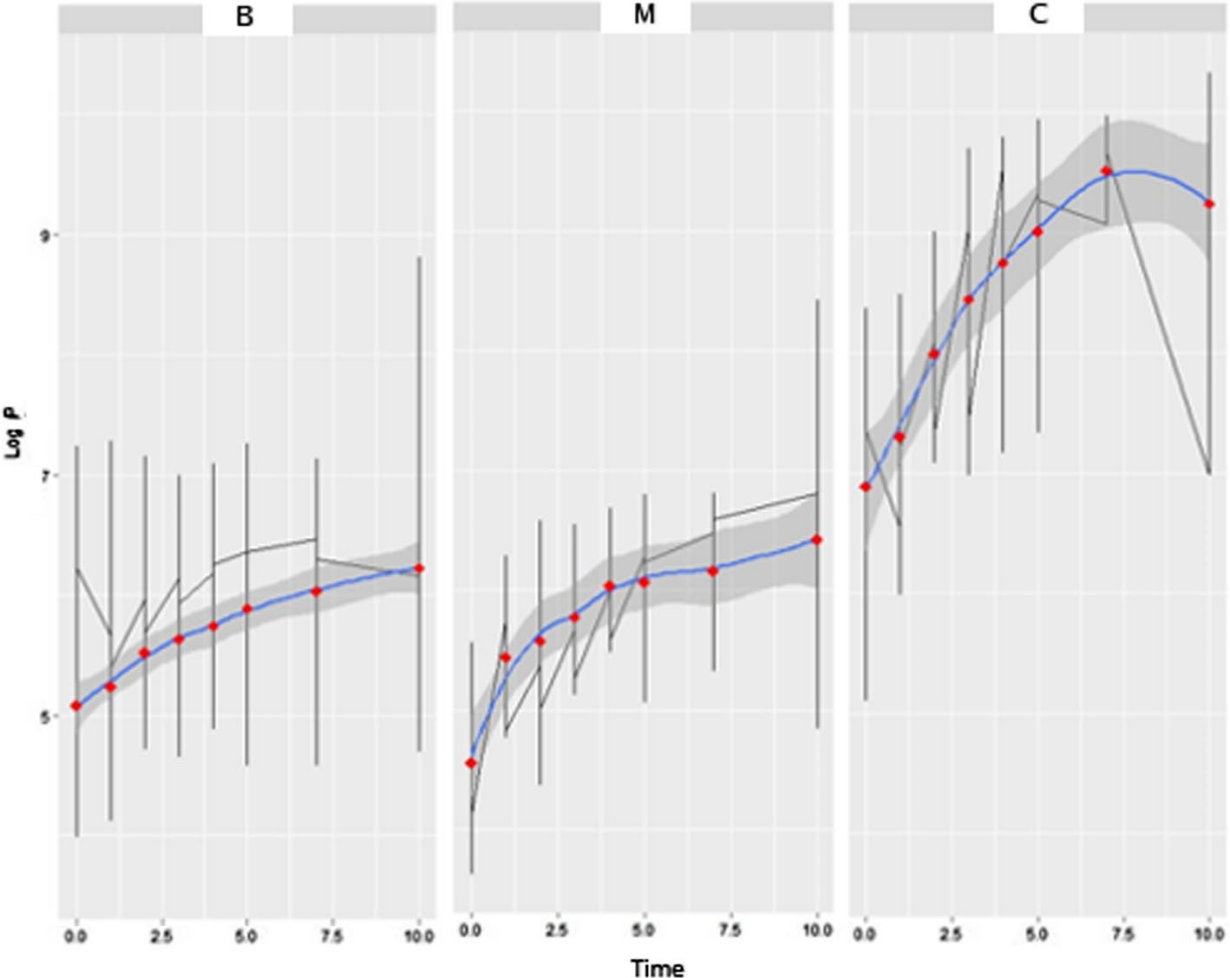
Thermal evolution of malignant, benign and control tumors over time. The random-effects model for longitudinal data (REM) was used and the differences between groups (**M**) for malignant, (**B**) for benign and (**C**) for control in the time of t_0_ = 0 to 10 min on the logarithmic pixel scale (LogP) were evaluated

Figure 2 illustrates the thermal rewarming in logarithmic pixel scale (LogP), with (M) for malignant nodule, (B) for benign nodule and (C) for control group.

We saw that in all simulations the value of the β0 intercept was always significant (*p* = 0.001), thus indicating that the beginnings of the rewarming period is essential for the analysis, and that the comparison of benign and malignant nodular areas is possible. Equation 1 demonstrates the model found:

1 $$ {\text{LogP }} = {5}.{9}0{167 } + \, 0.{19221 }*{\text{ time }} + \, \left( {{1}|{\text{ nodule}}} \right). $$

Applying the same model to the control group (healthy tissues), we can see that the time differs between them. The model values, considering the healthy plus the nodular tissues, are expressed in Eq. 2:

2 $$ {\text{LogP}} = {5}.{12825 } + \, 0.{15258 }*{\text{ time }} + \, \left( {{1}|{\text{ nodule}}} \right), $$

with β0 significance (p = 0.001).

Based on the longitudinal analysis, it is possible to notice that the intercept presents an important and significant element of distinction between the two types of nodules.

### Determination of thermal time interval

**Fig. 3:**
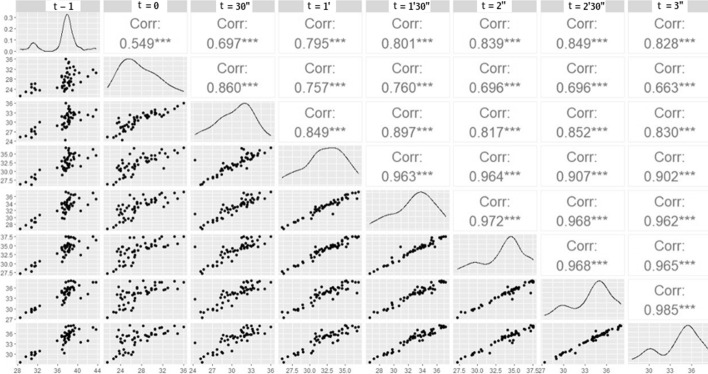
Scatter plot of samples over time. Evaluation of the correlation in time pre-stress to cold (t-1) until time 3 min (t3m) in a 30-s interval

**Fig. 4:**
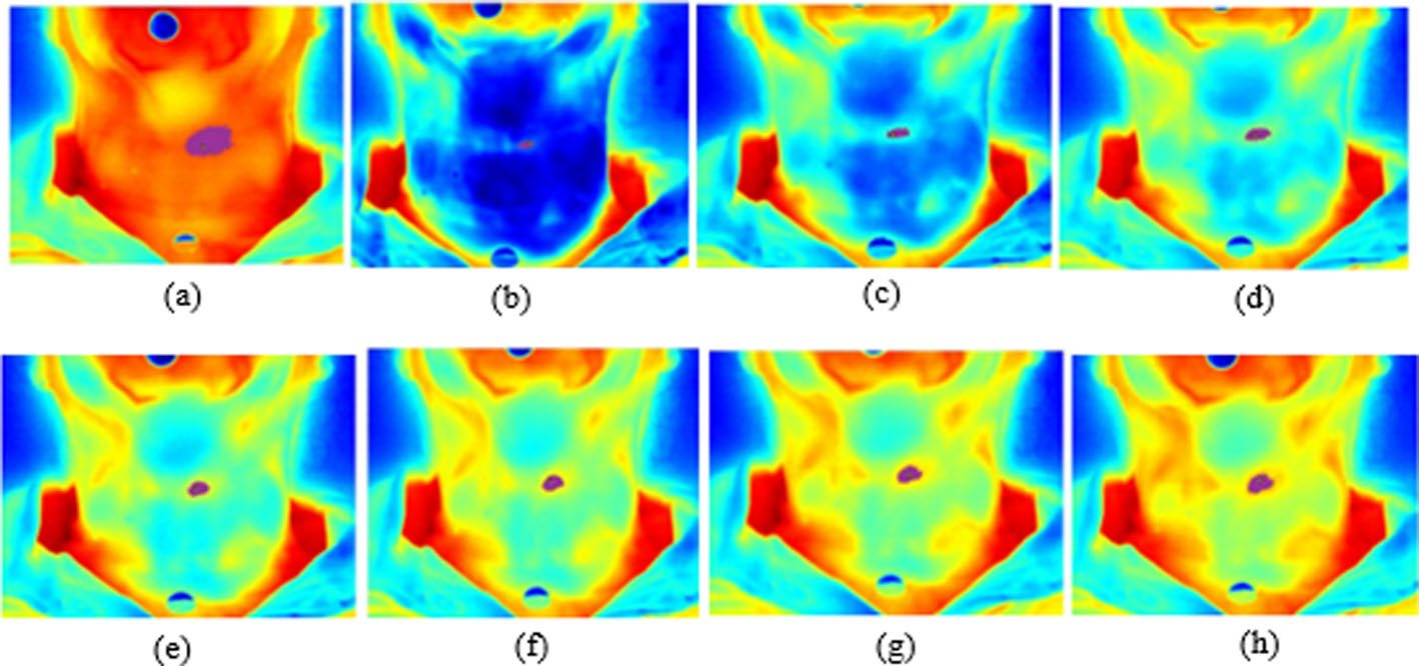
Thermographic image of a malignant tumor in the left lobe of the thyroid by segmentation. Region of interest (ROI) analyzed in the times: **a** t-1 (pre-cooling), **b** t_0_ = 0, **c** 30 s, **d** 1 min, **e** 1 min and 30 s, **f** 2 min, g 2 min and 30 s, **h** 3 min

To assess the correlation between the times, the scatter plot was used. It was observed that from pre-cold stress (*t*-1) to cooling (t_0 =_0), there is a low correlation of temperatures. Figure 3 shows the scatter plot image which demonstrates that over time the correlation increases, that is, the temperatures are very close to each other in the times above 3 min (*t* = 3). Thus, the time stipulated in the analysis was between time t-1 and time *t* = 3, illustrated in Fig. 4.

**Fig. 5:**
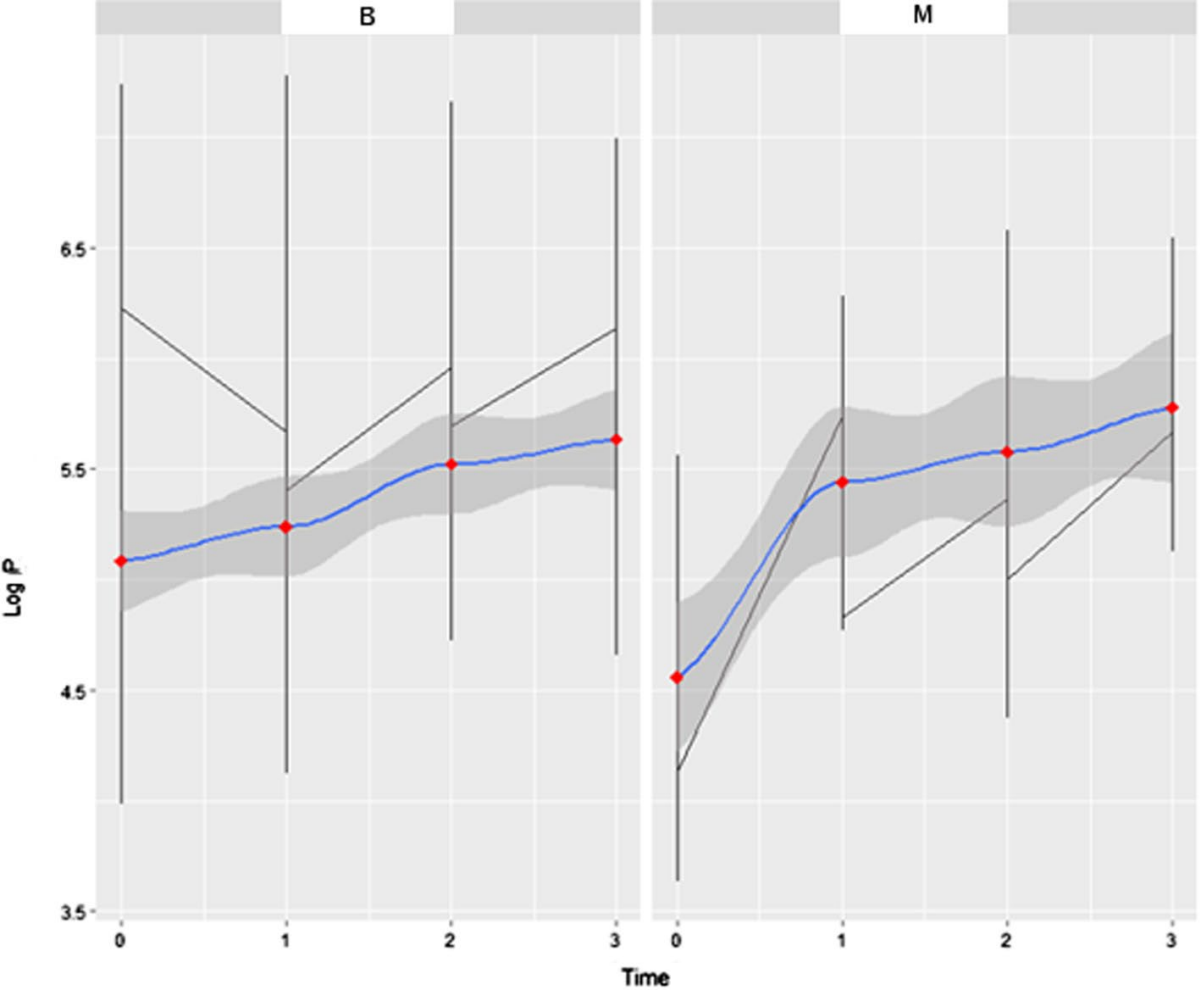
Evolution of pixel size in time 0 s to 3 min. Effect of rewarming of malignant (**M**) and benign (**B**) tissues on number of pixels on the logarithmic scale (LogP) in relation to time in minutes

By separating the data without including *t*-1, that is, t > 0, the warming of the region becomes evident. However, this time lapse is not sufficient to assess the rewarming effect of the malignant (M) and benign (B) tissues shown in Fig. 5.

As a result, the proposed fixed model was, Eq. 3:

3 $$ {\text{LogP }} = { 5}.{4}0{7587 } + \, 0.0{\text{76387 time}}|{\text{nodule}}{.} $$

The coefficients for the β0 intercept and the β1 coefficient for time are significant with *p*-value <  < 5%. That is, as time evolves, the number of pixels tends to increase at the following rate, Eq. 4:

4 $$ \frac{dLogp}{{dtime/nodule}} = 0.0763 \sim 7.6\% . $$

Overall, the result found is approximately 7.6% logarithmic growth rate in the number of pixels over time, which is significant.

### Inclusion of t-1

To assess the behavior of the thyroid gland from its normal thermal state to cooling, it is important to include time t-1. It was observed that the most relevant information is in the pre-stress subinterval (*t*-1) to the first 3 min (*t* = 3) immediately after cold stress. Thermally, cooling occurs between t -1 to its minimum, which is approximately t_0 =_0 and a time of 30 s for benign and malignant nodules, as evidenced by the analysis of the fixed temporal model for t < 0.

**Fig. 6:**
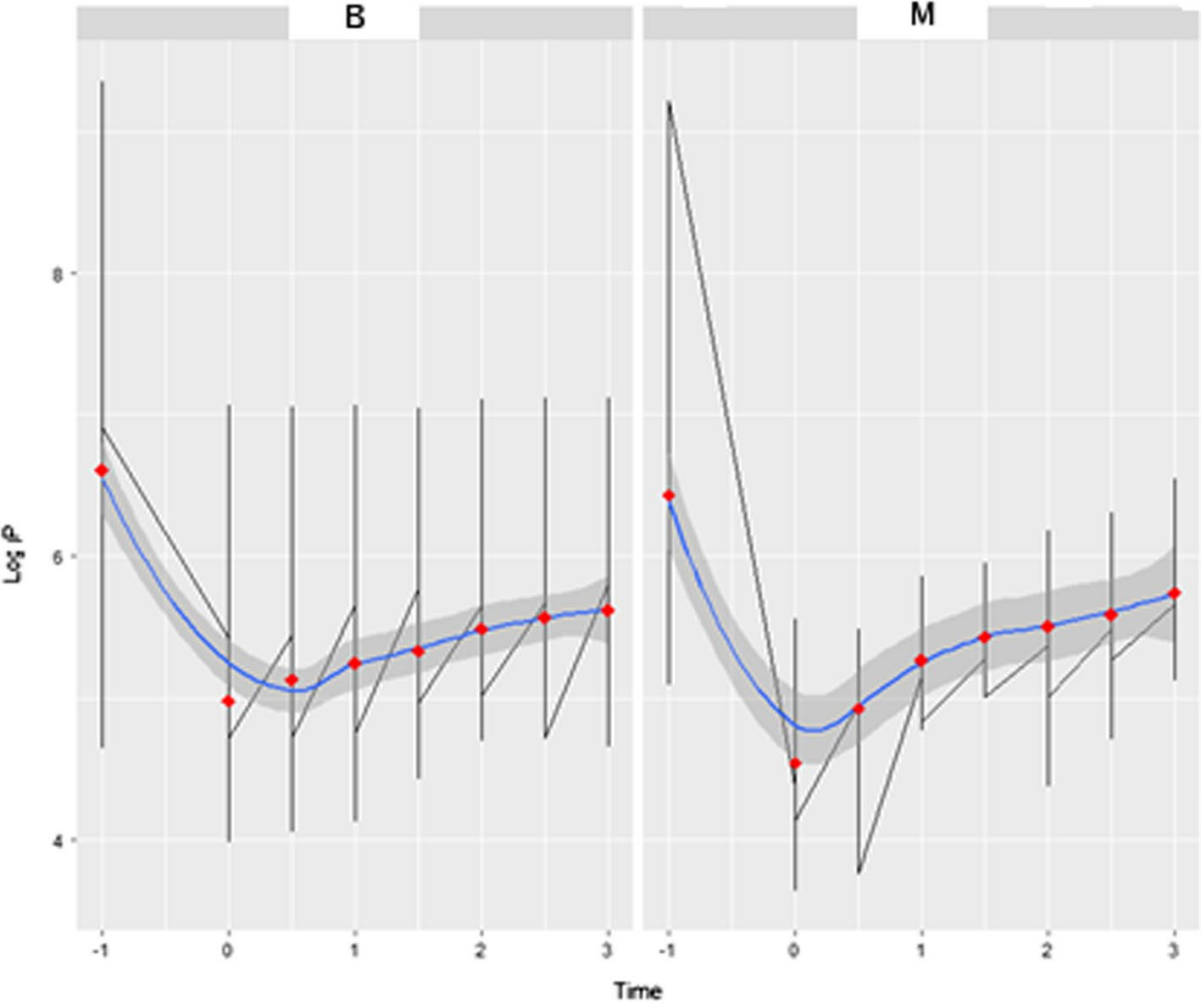
Longitudinal analysis in the pre-cooling time interval up to 3 min. The y-axis given by LogP and the x-axis, the time given in minutes in the 30-s interval, shows the difference between malignant (**M**) and benign (**B**) tissues in the time interval t (-1) to t (3), showing the “well” effect

In Fig. 6, the existence of a “well” effect is clear, or a global minimum that points to the beginnings of an increase in the quantity of pixels. The y-axis given by LogP and the x-axis, the time given in minutes in 30-s intervals, demonstrates the longitudinal analysis of malignant (M) and benign (B) tissues in the time interval t-1 to t = 3, evidencing the “well” effect.

This region, called “well”, includes times − 1 < t < 3 min, in 30 s intervals. There was a significant difference in the logarithmic scale in relation to the intercept (p < 0.001) and in the temporal evolution (*p* = 0.006).

This indicates a *p*-value <  < 5% (*p* = 0.001) for both the β0, intercept, as well as the β1 coefficient. This indicates that it is appropriate to use the region around the well (*p* = 0.001) to determine the effect of benign and malignant nodules on organic behavior during rewarming. Thus, this region is important in differentiating the malignant nodule from the benign nodule.

### Variations in data

Observing that the “well” behaves differently between tumors, it was initially evaluated for variability, using the F-Snedecor test. The test exhibited a variation ratio value of 1.816 between the benign and malignant values (*p* = 0.001). The variance of the benign nodule is 86% greater than that of the malignant nodule. When we compared the control group and the benign nodule, the variation ratio was 0.75. When we compared the control group and the malignant nodule, the variation ratio was 0.39.

## Discussion

The thermal behavior evaluated by dynamic thermography was fundamental for evaluation and conclusion of the results in our research. The statistical tests provided important information for creating a protocol model for thermal image acquisition. Longitudinal analysis indicated the beginnings of rewarming as essential for the analysis and comparison of tumor tissues (*p* = 0.001 for β0).

Accordingly, the cold stress method has been used in several studies because it allows for monitoring of the thermal recovery of tumor tissues [22–25]. In a study using cold stress protocol, the author states that the adoption of the protocol in the region of analysis makes thermal images less susceptible to the influence of environmental factors, which allows for the measuring of the temperature and physiological response of the tissues in a given period of time [24]. The same cold stress protocol was used in another study which added some precautions regarding the uniformity of the cooling agent, advising that a thermal image needs to be captured in order to verify the uniformity of color intensity in the thermogram [25].

The insertion of the pre-stress time was essential in verifying, from cooling to thermal recovery, the variation of tissues with nodules. We also noticed that benign and malignant tissues have different behavior in regard to thermal recovery. Studies report that the rate of blood perfusion in healthy tissue is different from that presented by tissue with nodules, after cold stress. Undamaged tissue tries to recover faster to external agents than cancer tissue, which already has increased vascularization, but an analysis to assess such changes was not performed [15, 21, 24].

Although there are no articles evaluating the thermal behavior of nodules in relation to time, a clinical study carried out in a patient with carcinoma in the left lobe of the thyroid shows the existence of an increasing rate of temperature variation over time [26].

In our study, the behavior of nodules was evaluated by the longitudinal model (fixed linear model) pointing out tissue changes over time and indicating that the image acquisition protocol proposed in this research was able to differentiate the nodules.

The research shows a tumor property around the “wells”—global minimum. The formation of the “well” indicates that the variability of frequency distribution is significantly different between malignant and benign nodules (*p* = 0.001). A study performed with breast tumors found a similar result to ours, showing that temperature changes around the malignant tumor tissue are greater than those around benign nodules or healthy tissue [27].

One of the limitations found in our research was the low sample number of volunteers, resulting in a low number of nodules. Enlargement of the sample would provide the refinement of statistical analysis techniques, such as Machine Learning, Deep Learning and Neural Networks. Another limitation of this work is the lack of covariates, such as thyroid function exams (TSH, T3 and T4). These complementary exams could help in the application of other longitudinal models that solicit covariates for better adjustment.

The 30-s time interval between the images taken limited the application of testing on the temperature volatility of malignant and benign tumors when compared to healthy tissues. Thus, collecting the thermal images at time intervals of less than 30 s, making use of filming and study in frames, is suggested, thus enabling the evaluation of the physical phenomenon of finding the “well” in order to obtain more information about the behavior of tumor tissues.

## Conclusion

Thermography has been described as a useful and promising tool to aid in the diagnosis of thyroid nodules associated with other complementary tests. The understanding of the thermal behavior generated by our research shows that a thermal imaging protocol should consider the application of cold stress, as this was fundamental to evaluating nodules; thus, allowing for the monitoring of the cooling and thermal recovery of tissues.

Furthermore, the longitudinal analysis used in this study showed that time is important and significant. It evidenced a temporal dependence between the thermal information. Assessing the correlation between the analysis times, through the scatter plot, the ideal defined time for image collection and analysis is 3 min, thus delimiting the collection time. The insertion of the pre-stress time was essential for verifying the variation of nodular tissues from cooling to recovery. 

Longitudinal analysis also indicated the beginnings of rewarming as essential for the analysis and comparison of malignant and benign tissues (*p* = 0.001 para β0).

Benign nodules showed high variability compared to malignant nodules. In relation to the control group, benign nodules showed greater variability when compared to the study group. Thus, it was found that benign nodules have a different thermal behavior than malignant nodules and that both have a different behavior than the control group.

### Methodology

This is an exploratory descriptive study with the objective of evaluating the thermal behavior of nodules in individuals with nodules in the thyroid gland through dynamic infrared thermography.

### Study population

*Individuals with suspected* thyroid gland neoplasm, treated at the Head and Neck Oncology Outpatient Clinic at the Hospital of Reference in the city of Curitiba, Brazil, participated in the study. The study was approved by the Research Ethics Committee and respects the content of Resolution nº 466/12, of the National Health Council of Brazil.

The inclusion criteria for the study group of this research were individuals of both sexes; adults with a minimum of 18 years of age who have or have not had nodules in the thyroid gland. A total of 33 people agreed to participate in the study (which amounted to 42 nodules, 11 being malignant and 31 benign). The control group consisted of nine (9) people who did not present any changes in the thyroid gland.

In the study group, the mean age of individuals with benign nodules was 52.7 years, of which 90% were women and 10% men. In individuals with malignant tumors, the mean age was 56.32 years, of which 81% were women and 19% men. It was observed that 84.84% of the individuals did not present any type of pathology in the thyroid gland before the tumor and 71% had no family history of neoplasia. In the control group, the mean age of the participants was 55.4 years, of which 66% were women and 34% men.

All volunteers filled out an anamnesis form and signed the Informed Consent Form and the Consent Form for the Use of Images.

### Thermal image acquisition protocol

For the acquisition of thermal images, a Fluke camera, model TI 32, was used, coupled to a Wide 1 lens (with a spatial resolution of 0.63 mrads). This camera model has a resolution of 76,800pixels, wavelength sensitivity of 7.5 µm to 13 µm, with thermal sensitivity less than or equal to 0.045ºC. The camera was fixed to a tripod and positioned vertically at 90 degrees from the neck. The distance between the neck and the camera lens was 1 m. At this distance the camera has an instantaneous field of view (IFOV) of 0.6 mm X 0.6 mm.

Parameters such as emissivity (ε = 0.98), temperature unit of measure (°C), background temperature (20 °C), transmission (100%) and lens selection were adjusted in-camera. We chose to use the “auto adjustment” mode to adjust the camera's focus, but when necessary, fine adjustments were performed manually.

All infrared image collection was carried out in a temperature-controlled room between 20 and 2 ºC, duly controlled by a digital thermometer. The room remained closed throughout the image acquisition time so that variations in temperature and humidity could be controlled, as well as air circulation within the environment.

**Fig.7:**
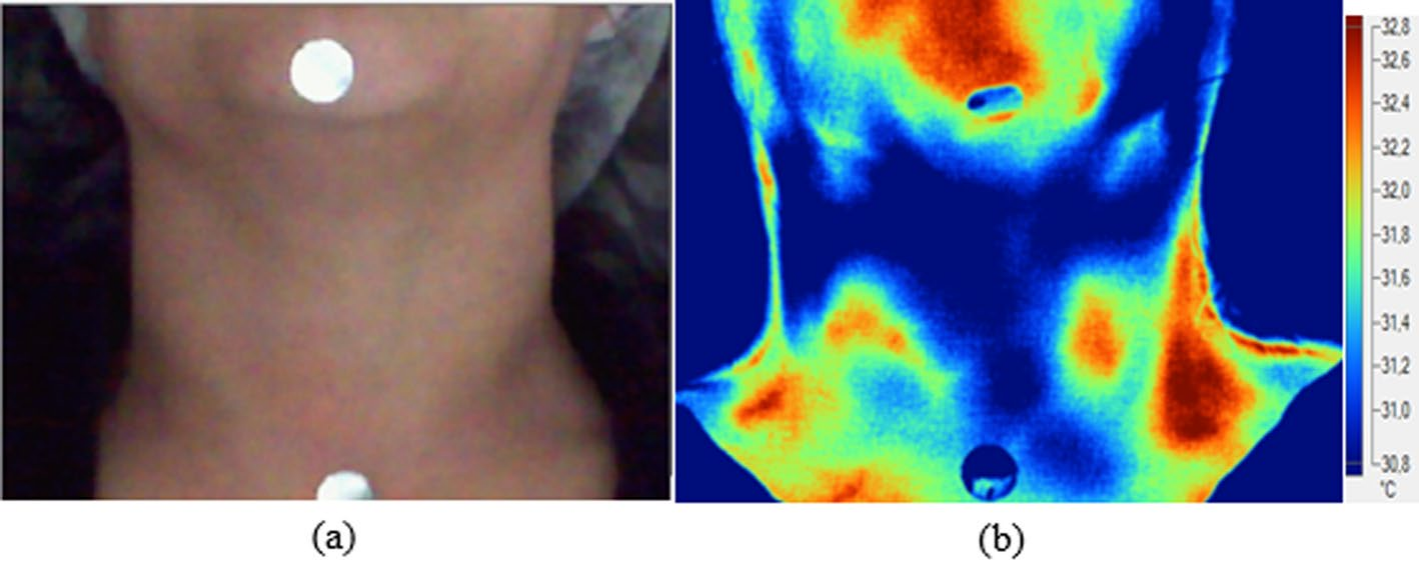
Image with metallic markers for delimiting the neck region. Markers in the chin protuberance region and in the sternal furcula region: **a** digital image **b** thermal image

For the acquisition of thermal images, the volunteers were in the lying position with their head in cervical extension supported on a cushion. Metallic markers were placed in the mentonian protuberance and in the region of the sternal notch, to delimit the region of analysis shown in Fig. 7.

**Fig. 8:**
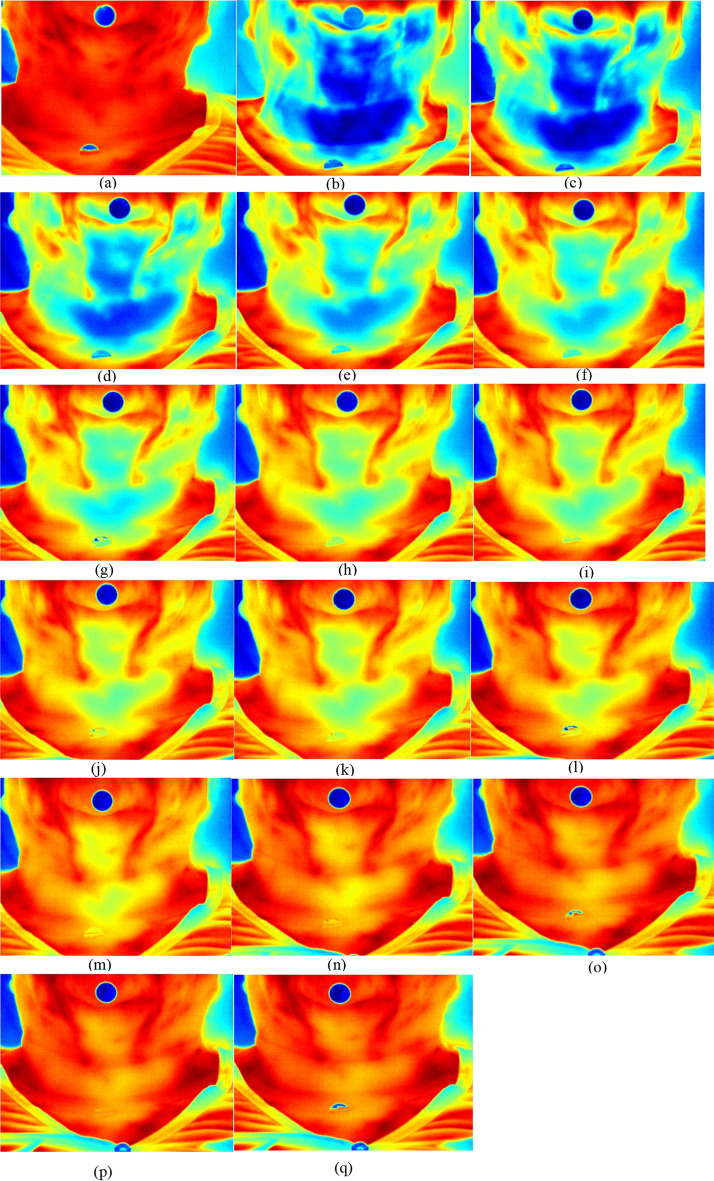
Dynamic thermographic image within 10 minutes. Thermographic images of the neck region (thyroid gland) at the following times: **a** pre-cooling time (t-1), **b** immediate post-cooling time (t_0 =_0), **c** 30 s, **d** 1 min, **e** 1 min and 30 s, **f** 2 min, **g** 2 min and 30 s, **h** 3 min, **i** 3 min and 30 s, **j** 4 min, **k** 4 min and 30 s, **l** 5 min, **m** 6 min, **n** 7 min, **o** 8 min, **p** 9 min and q 10 min

The first image (called t-1) was collected after acclimatization of the region (10 min with the region uncovered in the room at 20 ºC). Afterwards, cold stress was performed, with a gel pack cooled to 8 ºC, being placed across the neck, for 30 s. Image collections started immediate after removal of the gel pack, (t_0 =_0) extending for a period of *t *= 10 min. In the first five minutes, the images were taken every 30 s, and then afterwards, captured at an interval of one minute (Fig. 8).

### Thermal image processing

**Fig.9:**
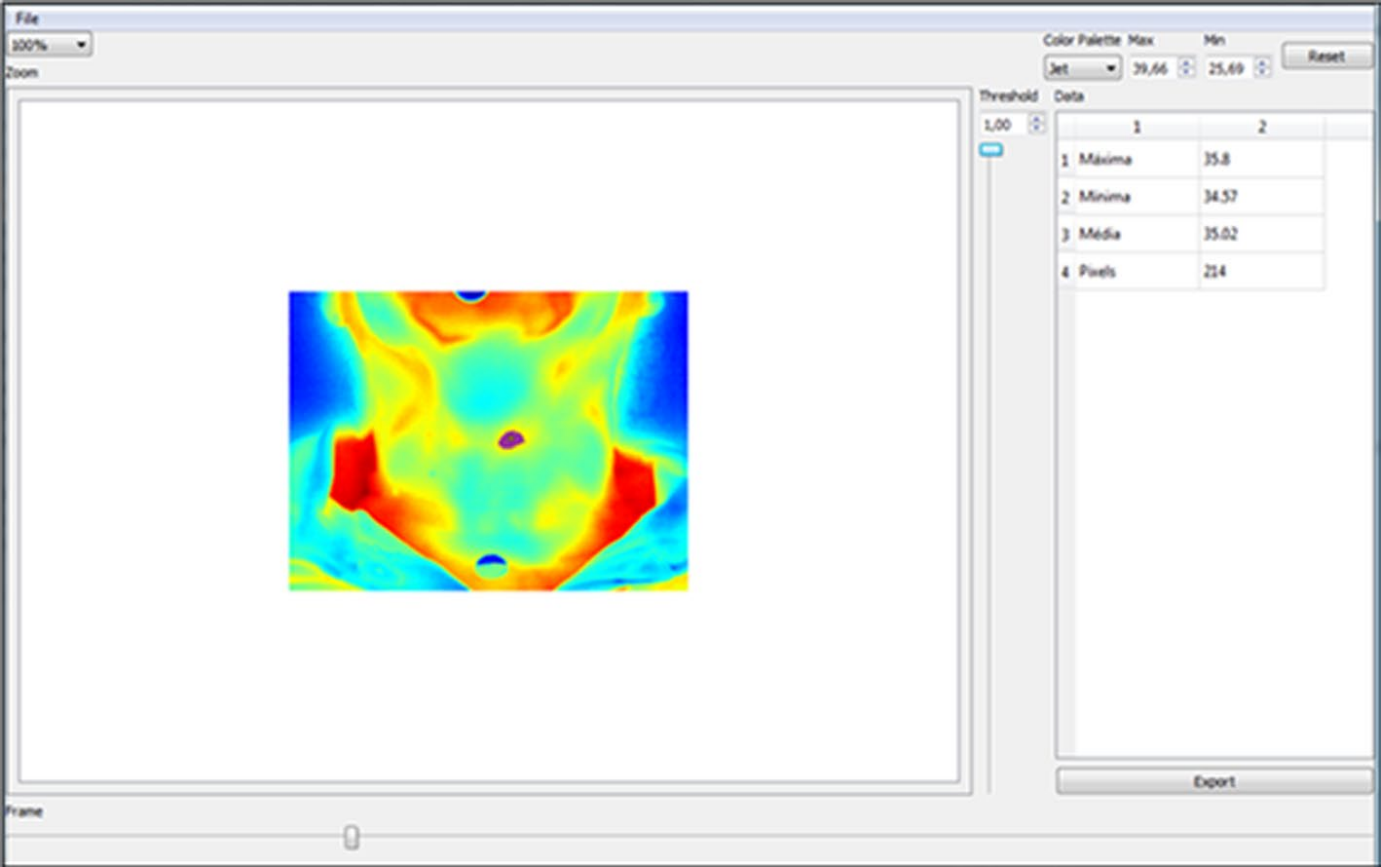
Thermographic image showing malignant cancer after processing the Python segmentation program. Thermographic image showing malignant cancer after processing the Python segmentation program. Region of interest (ROI) delimited by the program informing the minimum, average and maximum temperature and the number of pixels in the region with a nodule

The thermal images were initially processed using the SmartView 3.14 software (Fluke, USA), which allowed the choice of the temperature range and the red/blue color palette. Subsequently, in order to optimize the analysis of thermograms, a Python program capable of semi-automatically segmenting regions of interest was used. The program delimits the region of interest through pixels of the selected region. Delimited, the program showed the minimum, average and maximum temperature and the number of pixels in the region with nodule. Figure 9 demonstrates the demarcation, performed by the semi-automatic segmentation software, of a malignant nodule in the left lobe of the thyroid. Temporal evaluation measurements of nodular and healthy tissues were based on the quantity of pixel numbers in each current time period.

**Fig.10:**
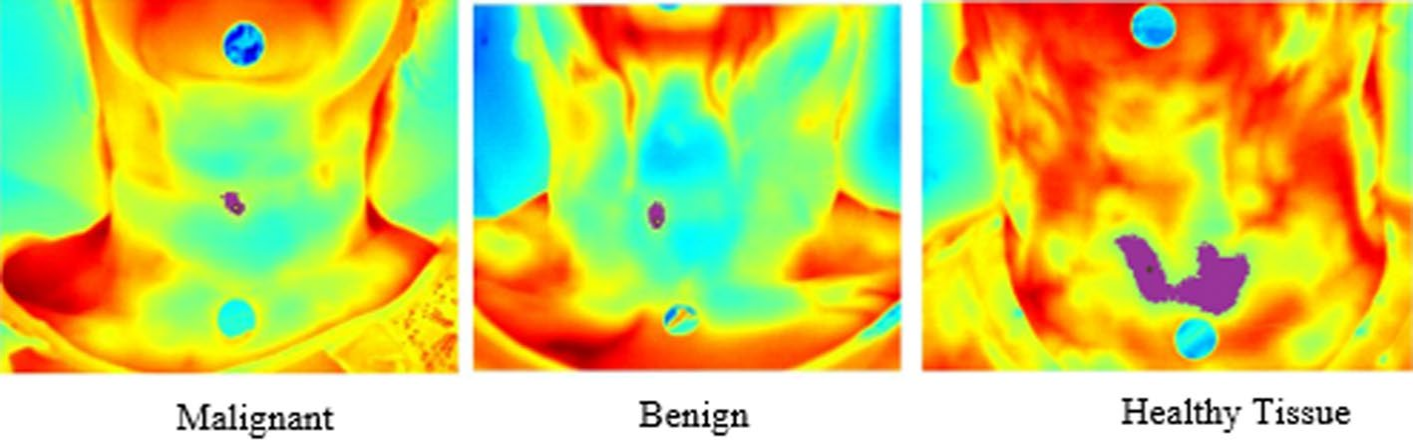
Time series graphs for the malignant, benign, and control

Thus, to increase the effectiveness of the analysis of this work, the region of interest was evaluated by the quantity of pixel numbers extracted from thermographic images by semi-automatic segmentation. Figure 10 shows the demarcation, performed by the semi-automatic segmentation software, of a malignant nodule, a benign nodule and healthy tissue (control group).

The delimitation of the area was based on ultrasound findings in order to assess the location and size of the nodule. The classification of the nodule was performed using the FNA exam which conveyed the nature of the lesion (benign or malignant). At the end of the work, 765 thermal images were evaluated.

### Statistical analysis

Firstly, an analysis was performed that evaluated the medians of thermal temperatures between tissue with a benign nodule and tissue with a malignant nodule. To verify the levels of thermal differences between the groups (control and healthy), the non-parametric Wilcoxon test was applied, transforming the information from the medians of minimum, average and maximum temperatures to pixels. The significance level was set at 95%. The second analysis aimed to measure the thermal recovery time between the nodules after cold stress.

The measures of interest are in relation to the temperatures of the neck, which represent the nodule region, the healthy region and core body temperature, respectively.

The temporal evaluation measures of the nodular and healthy tissues were based on the quantity of pixel numbers in logarithmic scale (LogP) in each current time period.

The research proposal led to the analysis of the longitudinal study to evaluate the thermal behavior of the nodules over time. Time was used as an independent variable to compose the equations of the linear models, and temperature was used as a dependent variable. The proposed models are represented by the multiple data regression given by Eq. 5:

5 $$ {\text{y }} = {\text{ X}}\beta + \varepsilon , $$

where the term y corresponds to the independent variable (time) with x being the dependent variable (or also known as covariate-temperature per pixel), with β the linear coefficients to be determined (intercept and slopes of an affine function). Observation error defined by ε follows a Gaussian distribution (N) with zero mean and variance σ2, expressed in Eq. 6:

6 $$ \varepsilon \sim {\text{N }}(0,\sigma {2}). $$

To assess the variation and behavior of the variability of the data, the F-Snedecor test and the Garch models were used, respectively.

Statistical analyses were performed using the R Core Team 2020 software.

## Supplementary Information

**Additional file 1: Table S1**In this study the value of ultrasound sensitivity, specificity, positive predictive value (PPV) and negative predictive value (NPV) values when compared to the biopsy result

## Data Availability

The data sets generated and/or analyzed during the present study are not publicly available based on the data access policy.

## Abbreviations

FNA: Fine needle aspiration
ROI: Region of interest
IFOV: Instantaneous field of view
REM: Random-effects model for longitudinal data
LogP: Pixel logarithmic scale
t-1: Pre-cooling time
t_0 =_0: Post-cooling time – 0 s
