# Supplementary material for: Thermography as an aid for the complementary diagnosis of nodules in the thyroid gland
Source: Biomed Eng Online. 2022 Jun 27;21:41. doi: 10.1186/s12938-022-01009-3 (PMC9235134; doi:10.1186/s12938-022-01009-3)
Supplement: Supplementary file 1 — Additional file 1: Table S1In this study the value of ultrasound sensitivity, specificity, positive predictive value (PPV) and negative predictive value (NPV) values when compared to the biopsy result [file 12938_2022_1009_MOESM1_ESM.docx]

Additional file Table S1 – In this study the value of ultrasound sensitivity, specificity, positive predictive value (PPV) and negative predictive value (NPV) values when compared to the biopsy result

|  | Malignant | Benign |  |
| --- | --- | --- | --- |
| Prevalence | 0.26 | 0.74 |  |
| Sensitivity | 0.82 | 0.52 |  |
| Specificity | 0.48 | 0.18 |  |
| Positive Predictive Value | 0.36 | 0.64 |  |
| Negative Predictive Value | 0.88 | 0.12 |  |

Author (2020)
